# Synthesis, crystal structure and properties of poly[di-μ_3_-chlorido-di-μ_2_-chlorido-bis­[4-methyl-*N*-(pyridin-2-yl­methyl­idene)aniline]dicadmium(II)]

**DOI:** 10.1107/S2056989024004274

**Published:** 2024-05-21

**Authors:** Chatphorn Theppitak, Sakchai Laksee, Kittipong Chainok

**Affiliations:** a Thammasat University Research Unit in Multifunctional Crystalline Materials and Applications (TU-MCMA), Faculty of Science and Technology, Thammasat University, Pathum Thani 12121, Thailand; b Office of Research, Chulabhorn Research Institute, Laksi, Bangkok 10210, Thailand; c Nuclear Technology Research and Development Center, Thailand Institute of Nuclear Technology (Public Organization), Nakhon Nayok 26120, Thailand; Vienna University of Technology, Austria

**Keywords:** crystal structure, coordination polymers, cadmium(II), Schiff base

## Abstract

The title coordination polymer with the 4-methyl-*N*-(pyridin-2-yl­methyl­idene)aniline Schiff base ligand (*L*, C_13_H_12_N_2_), [Cd_2_Cl_4_(C_13_H_12_N_2_)]_*n*_ (**1**), exhibits a columnar structure extending parallel to [100]. The columns are aligned in parallel and are decorated with chelating *L* ligands on both sides. They are elongated into a supra­molecular sheet extending parallel to (01  ) through π–π stacking inter­actions involving *L* ligands of neighbouring columns. Adjacent sheets are packed into the tri-periodic supra­molecular network through weak C—H⋯Cl hydrogen-bonding inter­actions that involve the phenyl CH groups and chlorido ligands. The thermal stability and photoluminescent properties of (**1**) have also been examined.

## Abstract 2

The bidentate *N*,*N*′-chelating Schiff base ligand *L* and cadmium(II) chloride create a self-assembled polymeric chain structure [Cd_2_Cl_4_(*L*)]_*n*_ comprising a defect cubane-like motif.

## 1. Chemical context

The design and construction of coordination polymers (CPs) have received continuous attention over the past three decades due to their intriguing functionalities (Batten *et al.*, 2008 ▸). These materials are assembled through the coordination bonds between metal ions and organic linkers, whereby their topologies and dimensionalities are highly dependent on synthetic parameters as well as the chemical nature of starting materials (Jiajaroen *et al.*, 2022 ▸; Li *et al.*, 2022 ▸). Among many others, CPs of group 12 metal ions have attracted great inter­est for their potential applications in photoluminescence and optoelectronics (Ren *et al.*, 2014 ▸; Shang *et al.*, 2020 ▸). In this context, organic linkers containing carboxyl­ates and/or nitro­gen heterocycles on their backbone have been widely used due to their abundant coordination sites when reacting with *d* ^10^ metal ions (Zhang *et al.*, 2020 ▸). On the other hand, inorganic halogenidometallates have also shown great potential as building blocks in various functional materials (Chen & Beatty, 2007 ▸; Zhai *et al.*, 2011 ▸; Freudenmann & Feldmann, 2014 ▸; Chen *et al.*, 2015 ▸). Specifically, chlorido­cadmate(II) anions are known to exist in various forms such as [CdCl_3_], [CdCl_4_], and [CdCl_6_] within different structural motifs (Gridley *et al.*, 2013 ▸; Mobin *et al.*, 2014 ▸; Wang *et al.*, 2017 ▸; Hu *et al.*, 2021 ▸). Notably, some of the corresponding materials exhibit high luminescence brightness (Zhai *et al.*, 2011 ▸).

In this work, a coordination polymer, [Cd_2_Cl_4_(*L*)]_*n*_ (**1**), has formed through self-assembly from CdCl_2_ and the 4-methyl-*N*-(pyridin-2-yl­methyl­idene)aniline (*L*) Schiff base ligand. Next to the structural set-up, the thermal stability and solid-state photoluminescence properties of (**1**) were investigated and are discussed in detail. 
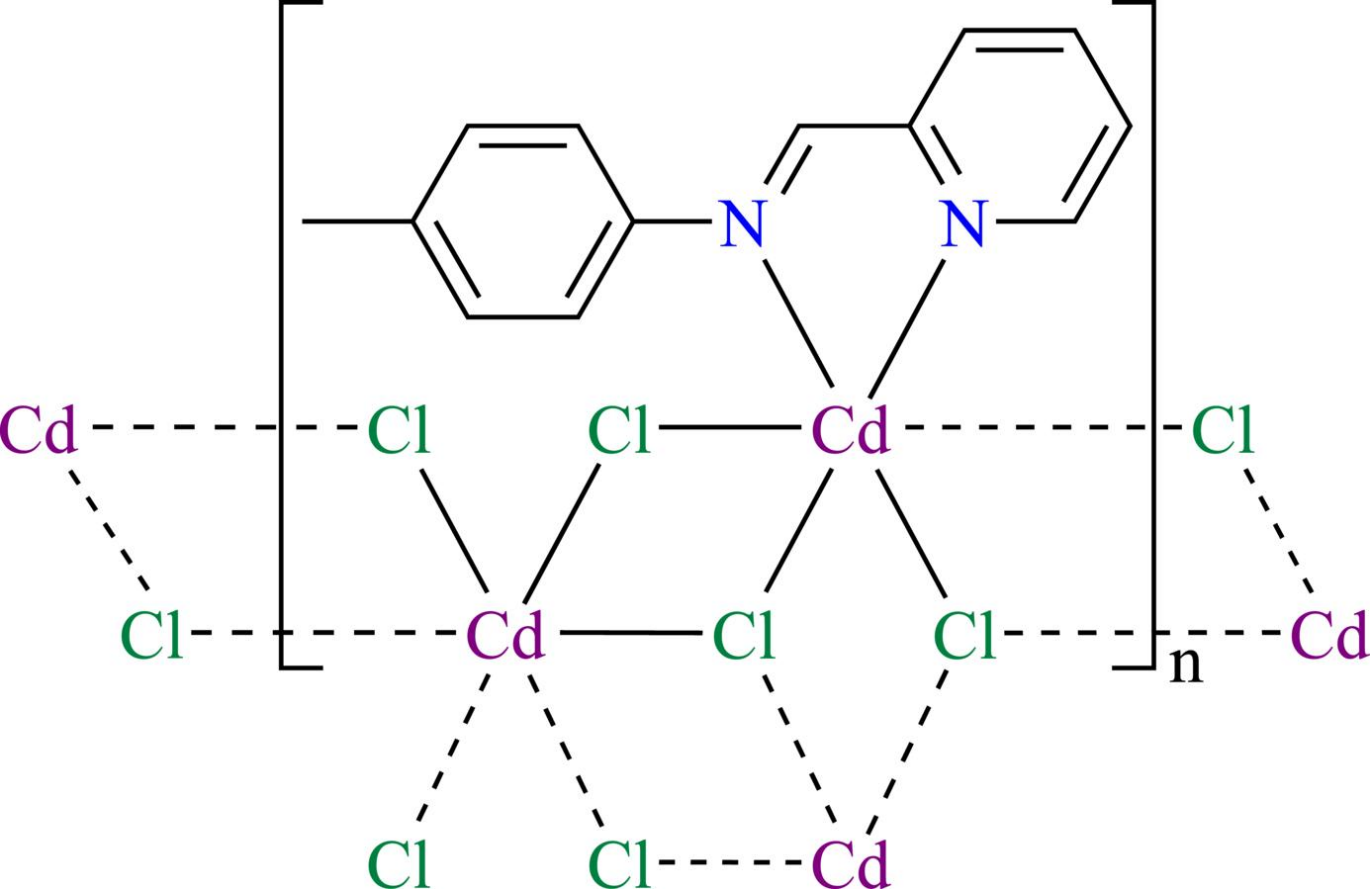


## 2. Structural commentary

**Figure 1:**
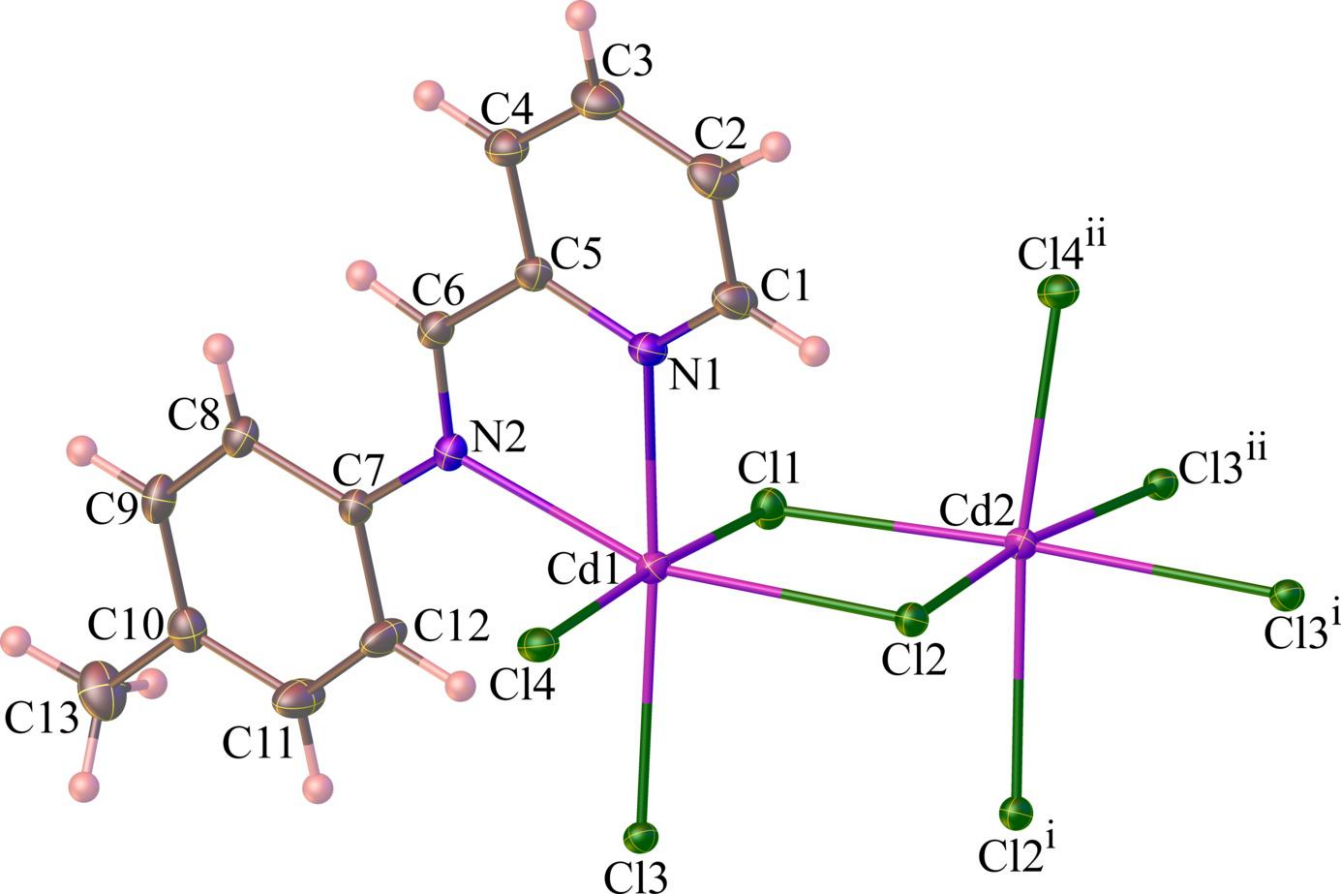
The expanded asymmetric unit of (**1**) showing the full coordination spheres of the two Cd^II^ atoms. Displacement ellipsoids are drawn at the 30% probability level. [Symmetry codes: (i) −*x*, −*y*, −*z*; (ii) *x* − 1, *y*, *z*.]

The asymmetric unit of (**1**) contains two Cd^II^ atoms, one Schiff base ligand *L*, and four chlorido ligands. Both Cd1 and Cd2 have a distorted octa­hedral coordination environment. As depicted in Fig. 1 ▸, Cd1 displays a [Cl_4_N_2_] coordination set defined by two *μ*_3_-Cl atoms in the equatorial plane, two *μ*_2_-Cl atoms in the axial positions and two *N* atoms from the chelating ligand *L* in the remaining equatorial sites, whereas Cd2 is in a [Cl_6_] coordination set by two *μ*_2_- and four *μ*_3_-Cl atoms. The corresponding bond angles around the central Cd1 and Cd2 atoms vary from 72.51 (12) to 176.14 (3)° and 81.21 (3) to 176.84 (3)°, respectively. The Cd—Cl bond lengths are in the range 2.5729 (10) – 2.7555 (10) Å, expectedly longer than those of the Cd—N bonds (2.311 (3) and 2.378 (3) Å). These values are in the normal range reported for related Cd^II^ compounds (Zhai *et al.*, 2011 ▸).

**Figure 2:**
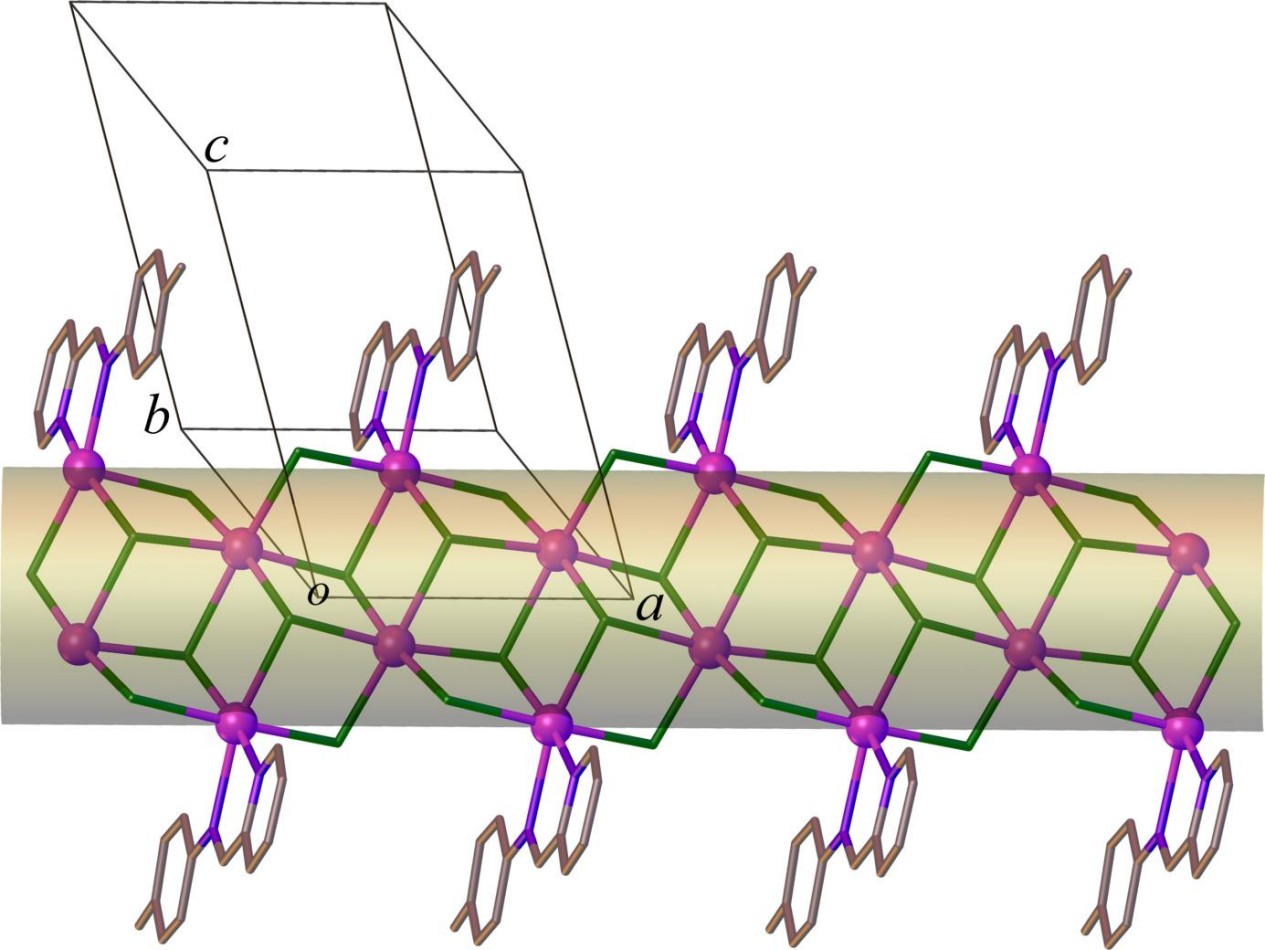
The columnar structure of (**1**) extending parallel to [100]. Hydrogen atoms are omitted for clarity.

The *μ*_2_- and *μ*_3_-bridging character of the chlorido ligands leads to a columnar motif with composition [Cd_2_Cl_4_(*L*)]_*n*_ running parallel to [100], as shown in Fig. 2 ▸. The columns contain a cubane-like [Cd_2_Cl_4_] unit with a missing vertex with diagonal Cd⋯Cd separations in the range from 3.853 (3) to 3.973 (3) Å. The chelating ligands *L* are arranged on both sides of the column motif.

## 3. Supra­molecular features

**Figure 3:**
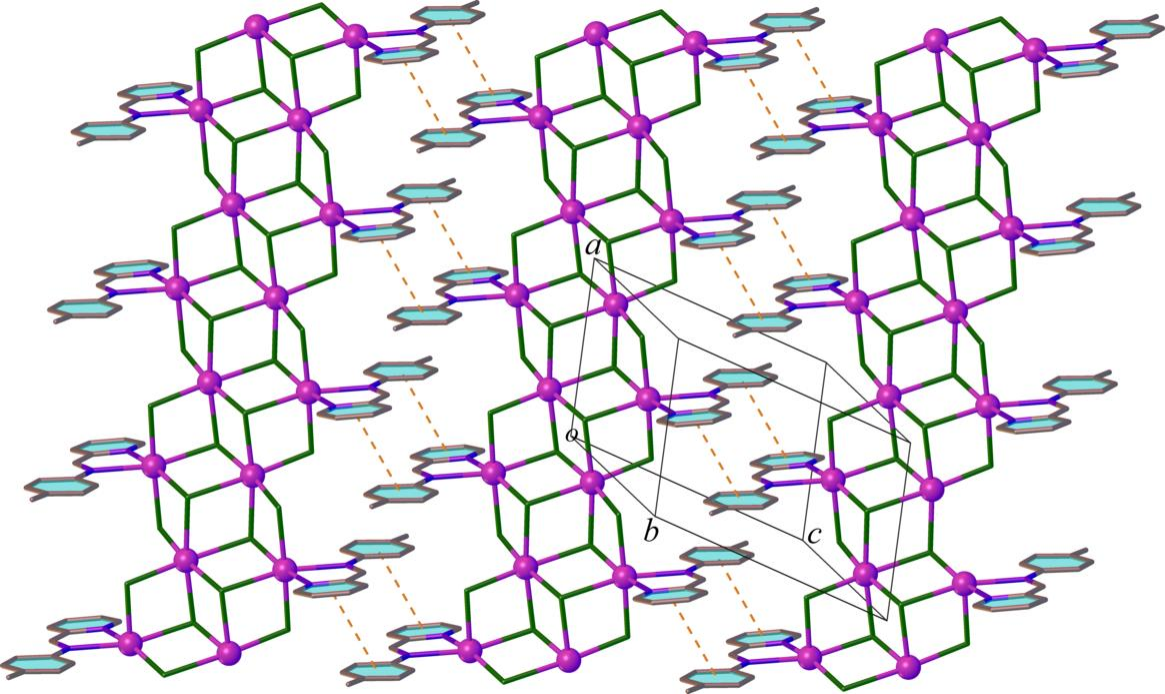
A view of the supra­molecular sheet structure in (**1**) with π–π inter­actions shown as dashed lines. Hydrogen atoms are omitted for clarity.

In the crystal, ligands *L* inter­act with those from neighbouring columns through π–π stacking inter­actions, where parallel planes of phen­yl/pyridyl rings are slightly offset (slippage 1.518 and 1.810 Å) with a centroid-to-centroid distance of 3.700 (3) Å and a dihedral angle of 5.61 (3)°. This arrangement leads to the formation of supra­molecular sheets extending parallel to (01 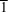 ) (Fig. 3 ▸). There is also a weak C—H⋯Cl hydrogen bond between the phenyl CH group and a chlorido ligand in adjacent columns [C9—H9⋯Cl4^(i)^ = 3.552 (2) Å, C9—H9⋯Cl4^(i)^ = 146°, symmetry code: (i) 2 − *x*, 1 − *y*, 1 − *z*). The sheets are connected by additional C—H⋯Cl hydrogen bonds (C2—H2⋯Cl4^(ii)^ = 3.697 (3) Å, C2—H2⋯Cl4^(ii)^ = 159°, symmetry code: (ii) 1 − *x*, 1 − *y*, −*z*), resulting in a tri-periodic supra­molecular structure. It is noteworthy that no significant Cl⋯Cl halogen-bonding inter­actions occur. This likely is a result of the bidentate *L* ligands establishing steric hindrance within the coordination sphere of the Cd1 atom.

## 4. Powder X-ray diffraction (PXRD) and thermogravimetry (TG)

**Figure 4:**
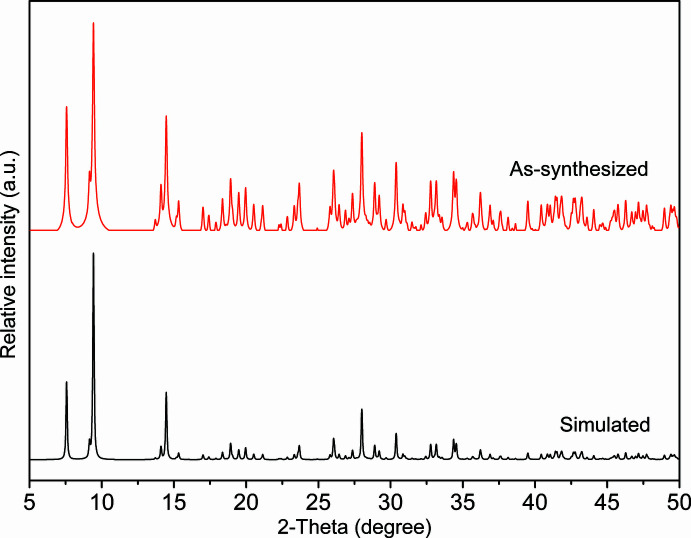
Comparison of experimental and simulated PXRD patterns of (**1**) at room temperature.

The phase purity of (**1**) was revealed by room-temperature PXRD measurements with a good match between experimental and simulated peak positions (Fig. 4 ▸). It should be noted that the differences in the intensity may be due to preferred orientation of the crystallites in the sample.

**Figure 5:**
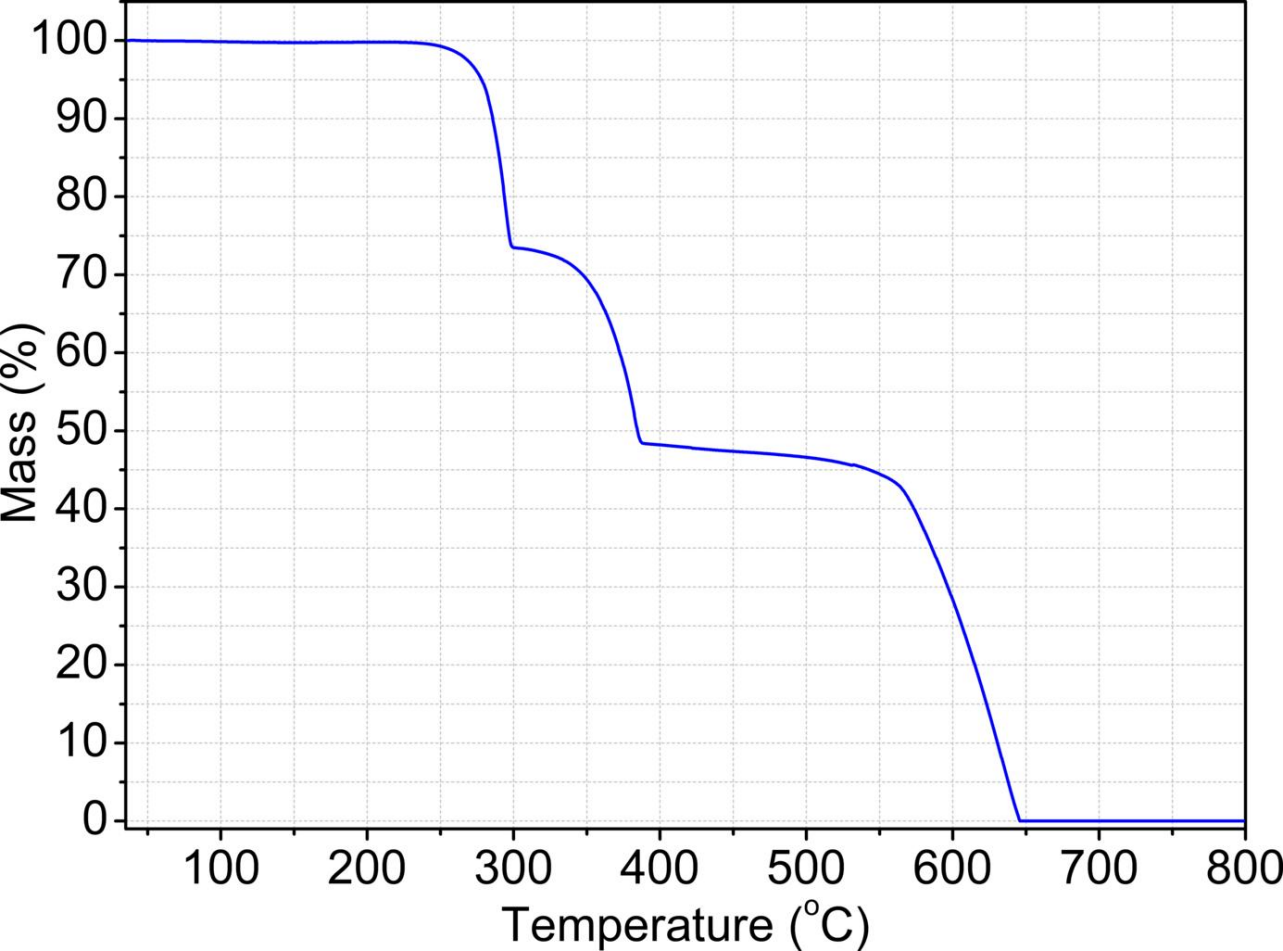
TG curve of (**1**).

The thermal stability of (**1**) was studied by TG measurements. As can be seen in Fig. 5 ▸, the TG curve of (**1**) shows three consecutive steps of mass loss in the range of 530–920 K. However, these steps cannot be assigned clearly. There is no mass loss from room temperature to 520 K, indicating that solvent mol­ecules are not incorporated.

## 5. Solid-state photoluminescence properties

**Figure 6:**
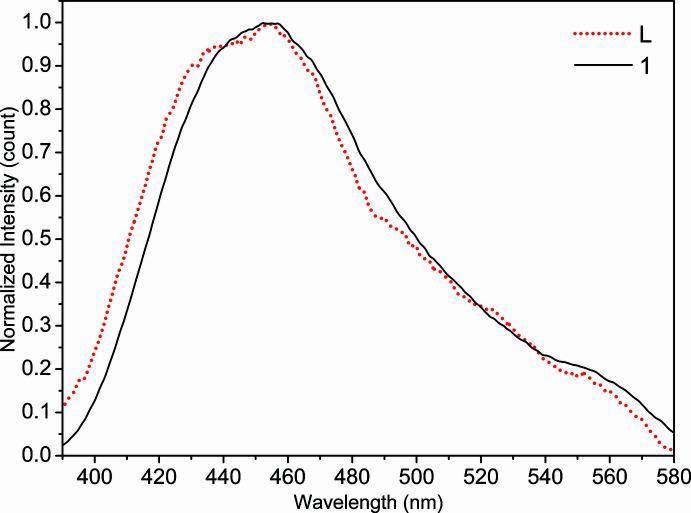
The solid-state photoluminescence spectra of ligand *L* and (**1**) at room temperature.

The solid-state photoluminescence spectra of the Schiff base ligand *L* and coordination polymer (**1**) were recorded at room temperature (Fig. 6 ▸). Upon excitation at 325 nm, the free ligand *L* displays a broad blue fluorescent emission at 456 nm, while (**1**) exhibits photoluminescence with a maximum at 457 nm upon excitation at 340 nm. Because metal ions with *d* ^10^ configuration usually are stable, the luminescence of complex (**1**) can solely be attributed to the intra-ligand *π* → π* emission state (*i.e.* ligand-based emission), which is also found in the free ligand *L* itself (Zhao *et al.*, 2017 ▸).

## 6. Database survey

A search of the Cambridge Structural Database (CSD, version 5.44, last update in April 2023; Groom *et al.*, 2016 ▸) using the ConQuest software (Bruno *et al.*, 2002 ▸) yielded 17 hits for a fragment of a chlorido-bridged tetra­nuclear cadmium(II) compound with a defect cubane-like core. There are two mono-periodic coordination polymers that include organic ligands organised on both sides of the chain motif, similar to the arrangement in (**1**), *viz*. IQATAY (Hu *et al.*, 2021 ▸) and SOGREN (Biet & Avarvari, 2014 ▸). In addition, 50 complexes of the title Schiff base ligand 4-methyl-*N*-(pyridin-2-yl­methyl­idene)aniline appear in the CSD. All these complexes are mononuclear with the Schiff base ligands acting in a bidentate chelating fashion. In the crystal packing of these compounds, π–π stacking and weak C—H⋯π inter­actions are frequently observed.

## 7. Synthesis and crystallization

A solution of 4-methyl-*N*-(2-pyridyl­methyl­ene)aniline (61.6 mg, 0.2 mmol) in dry di­chloro­methane (2 ml) was placed in a test tube. A mixture of aceto­nitrile and di­chloro­methane solution (6 ml, 1:1, *v*/*v*) was carefully added on the top. A solution of CdCl_2_·6H_2_O (19.8 mg, 0.2 mmol) in dry aceto­nitrile (2 ml) was then carefully layered on the top of the aceto­nitrile/di­chloro­methane mixed solution. After slow diffusion at room temperature for a week, light-yellow block-shaped crystals of (**1**) were obtained. Yield: 57% based on Cd. Analysis calculated for C_13_H_12_Cd_2_Cl_4_N_2_: C, 27.74; H, 2.15; N, 4.98%; found: C, 27.69; H, 2.18; N, 4.72%. IR (ATR mode, cm^−1^): 3027 (*w*), 2943 (*w*), 1899 (*w*), 1590 (*m*), 1504 (*m*), 1441 (*m*), 1268 (*m*), 1158 (*m*), 1015 (*m*), 908 (*m*), 817 (*s*), 781 (*s*), 638 (*m*), 539 (*s*), 412 (*m*).

**Experimental details**

All commercially available chemicals and solvents were of reagent grade and were used as received without further purification. Elemental (C, H, N) analysis was performed on a LECO CHNS 932 elemental analyser. IR spectra were recorded on a Bruker model INVENIO R spectrometer using ATR mode, in the range of 650–4000 cm^−1^. PXRD measurements were performed on a Bruker D2 Phaser X-ray diffractometer equipped with graphite monochromatized Cu *K*α radiation (λ = 1.54056 Å) at 30 kV and 10 mA. Simulated PXRD pattern were calculated from single-crystal X-ray diffraction data and processed with Mercury (Macrae *et al.*, 2020 ▸). The TG measurements were performed in an N_2_ atmosphere on a TGA 55 TA Instrument from ambient temperature up to 1223 K with a heating rate of 10 K min^−1^. The solid-state photoluminescence spectra were measured at room temperature using a Horiba Scientific model FluoroMax-4 spectro­fluoro­meter.

## 8. Refinement

Crystal data, data collection, and structure refinement details are summarized in Table 1 ▸. The carbon-bound H atoms were placed in geometrically calculated positions and refined as riding with C—H = 0.93 Å and *U*_iso_(H) = 1.2*U*_eq_(C).

## Supplementary Material

Crystal structure: contains datablock(s) I. DOI: 10.1107/S2056989024004274/wm5718sup1.cif

Structure factors: contains datablock(s) I. DOI: 10.1107/S2056989024004274/wm5718Isup2.hkl

Supporting information file. DOI: 10.1107/S2056989024004274/wm5718Isup3.cdx

CCDC reference: 2354120

Additional supporting information:  crystallographic information; 3D view; checkCIF report

## Figures and Tables

**Table 1:** Experimental details

Crystal data	
Chemical formula	[Cd_2_Cl_4_(C_13_H_12_N_2_)]
*M*_r_	562.85
Crystal system, space group	Triclinic, *P* 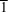
Temperature (K)	296
*a*, *b*, *c* (Å)	6.8597 (2), 10.8855 (4), 12.8106 (5)
α, β, γ (°)	107.566 (1), 100.523 (1), 106.799 (1)
*V* (Å^3^)	834.02 (5)
*Z*	2
Radiation type	Mo *K*α
μ (mm^−1^)	3.18
Crystal size (mm)	0.18 × 0.14 × 0.14

Data collection	
Diffractometer	Bruker D8 QUEST CMOS PHOTON II
Absorption correction	Multi-scan (*SADABS*; Krause *et al.*, 2015 ▸)
*T*_min_, *T*_max_	0.696, 0.745
No. of measured, independent and observed [*I* > 2σ(*I*)] reflections	20153, 3418, 2553
*R*_int_	0.050
(sin θ/λ)_max_ (Å^−1^)	0.625

Refinement	
*R*[*F* ^2^ > 2σ(*F* ^2^)], *wR*(*F* ^2^), *S*	0.030, 0.066, 1.03
No. of reflections	3418
No. of parameters	191
H-atom treatment	H-atom parameters constrained
Δρ_max_, Δρ_min_ (e Å^−3^)	0.73, −0.41

Computer programs: *APEX4* and *SAINT* (Bruker, 2019 ▸), *SHELXT2014/4* (Sheldrick, 2015*a* ▸), *SHELXL2018/3* (Sheldrick, 2015*b* ▸) and *OLEX2* (Dolomanov *et al.*, 2009 ▸).

## supplementary crystallographic information

### Crystal data

**Table:**

[Cd_2_Cl_4_(C_13_H_12_N_2_)]	*Z* = 2
*M**_r_* = 562.85	*F*(000) = 536
Triclinic, *P*1	*D*_x_ = 2.241 Mg m^−^^3^
*a* = 6.8597 (2) Å	Mo *K*α radiation, λ = 0.71073 Å
*b* = 10.8855 (4) Å	Cell parameters from 1412 reflections
*c* = 12.8106 (5) Å	θ = 3.2–25.2°
α = 107.566 (1)°	µ = 3.18 mm^−^^1^
β = 100.523 (1)°	*T* = 296 K
γ = 106.799 (1)°	Block, yellow
*V* = 834.02 (5) Å^3^	0.18 × 0.14 × 0.14 mm

### Data collection

**Table:**

Bruker D8 QUEST CMOS PHOTON II diffractometer	3418 independent reflections
Radiation source: sealed x-ray tube, Mo	2553 reflections with *I* > 2σ(*I*)
Graphite monochromator	*R*_int_ = 0.050
Detector resolution: 7.39 pixels mm^-1^	θ_max_ = 26.4°, θ_min_ = 3.2°
ω and φ scans	*h* = −8→8
Absorption correction: multi-scan (SADABS; Krause *et al.*, 2015)	*k* = −13→13
*T*_min_ = 0.696, *T*_max_ = 0.745	*l* = −15→15
20153 measured reflections	

### Refinement

**Table:**

Refinement on *F*^2^	Primary atom site location: dual
Least-squares matrix: full	Hydrogen site location: inferred from neighbouring sites
*R*[*F*^2^ > 2σ(*F*^2^)] = 0.030	H-atom parameters constrained
*wR*(*F*^2^) = 0.066	*w* = 1/[σ^2^(*F*_o_^2^) + (0.0267*P*)^2^ + 0.7296*P*] where *P* = (*F*_o_^2^ + 2*F*_c_^2^)/3
*S* = 1.03	(Δ/σ)_max_ < 0.001
3418 reflections	Δρ_max_ = 0.73 e Å^−^^3^
191 parameters	Δρ_min_ = −0.41 e Å^−^^3^
0 restraints	

### Special details

**Table:**

Geometry. All esds (except the esd in the dihedral angle between two l.s. planes) are estimated using the full covariance matrix. The cell esds are taken into account individually in the estimation of esds in distances, angles and torsion angles; correlations between esds in cell parameters are only used when they are defined by crystal symmetry. An approximate (isotropic) treatment of cell esds is used for estimating esds involving l.s. planes.

### Fractional atomic coordinates and isotropic or equivalent isotropic displacement parameters (Å^2^)

**Table:**

	*x*	*y*	*z*	*U*_iso_*/*U*_eq_	
Cd1	0.43896 (5)	0.25775 (3)	0.19523 (2)	0.03039 (10)	
Cd2	−0.17441 (5)	0.09278 (3)	0.07673 (2)	0.03114 (10)	
Cl1	0.11124 (16)	0.18034 (11)	0.27038 (9)	0.0364 (3)	
Cl3	0.46812 (15)	0.00951 (10)	0.13602 (8)	0.0274 (2)	
Cl4	0.76831 (17)	0.31838 (11)	0.11374 (9)	0.0369 (3)	
Cl2	0.15327 (15)	0.15624 (10)	−0.01197 (8)	0.0294 (2)	
N1	0.4732 (6)	0.4839 (3)	0.2237 (3)	0.0345 (8)	
N2	0.6415 (5)	0.3967 (3)	0.3893 (3)	0.0298 (8)	
C1	0.4082 (8)	0.5310 (5)	0.1449 (4)	0.0456 (12)	
H1	0.322184	0.467069	0.072599	0.055*	
C2	0.4618 (8)	0.6699 (5)	0.1650 (4)	0.0522 (13)	
H2	0.415590	0.698893	0.107078	0.063*	
C3	0.5849 (8)	0.7646 (5)	0.2725 (5)	0.0525 (13)	
H3	0.622400	0.859025	0.288606	0.063*	
C4	0.6520 (7)	0.7183 (5)	0.3561 (4)	0.0424 (11)	
H4	0.733459	0.780776	0.429718	0.051*	
C5	0.5963 (6)	0.5771 (4)	0.3286 (3)	0.0322 (10)	
C6	0.6732 (6)	0.5254 (4)	0.4146 (4)	0.0334 (10)	
H6	0.746876	0.587535	0.489373	0.040*	
C7	0.7158 (6)	0.3456 (4)	0.4728 (3)	0.0303 (9)	
C8	0.8273 (7)	0.4274 (5)	0.5868 (4)	0.0365 (10)	
H8	0.863455	0.522899	0.613147	0.044*	
C9	0.8839 (7)	0.3655 (5)	0.6609 (4)	0.0416 (11)	
H9	0.959473	0.421358	0.736998	0.050*	
C10	0.8331 (7)	0.2238 (5)	0.6266 (4)	0.0427 (11)	
C11	0.7295 (9)	0.1463 (5)	0.5128 (4)	0.0563 (14)	
H11	0.700061	0.051484	0.485576	0.068*	
C12	0.6677 (8)	0.2051 (5)	0.4378 (4)	0.0512 (14)	
H12	0.591889	0.148618	0.361789	0.061*	
C13	0.8854 (9)	0.1584 (6)	0.7100 (5)	0.0617 (15)	
H13A	0.989813	0.228147	0.779111	0.093*	
H13B	0.941330	0.089598	0.676953	0.093*	
H13C	0.758629	0.115638	0.727315	0.093*	

### Atomic displacement parameters (Å^2^)

**Table:**

	*U*^11^	*U*^22^	*U*^33^	*U*^12^	*U*^13^	*U*^23^
Cd1	0.03240 (18)	0.02790 (18)	0.02645 (17)	0.01212 (14)	0.00518 (13)	0.00540 (13)
Cd2	0.02639 (17)	0.03096 (18)	0.03128 (19)	0.01317 (14)	0.00606 (14)	0.00424 (14)
Cl1	0.0329 (6)	0.0427 (6)	0.0300 (5)	0.0128 (5)	0.0076 (4)	0.0107 (5)
Cl3	0.0302 (5)	0.0259 (5)	0.0252 (5)	0.0115 (4)	0.0081 (4)	0.0075 (4)
Cl4	0.0367 (6)	0.0292 (6)	0.0432 (6)	0.0126 (5)	0.0138 (5)	0.0096 (5)
Cl2	0.0305 (5)	0.0300 (5)	0.0262 (5)	0.0126 (4)	0.0056 (4)	0.0087 (4)
N1	0.039 (2)	0.031 (2)	0.036 (2)	0.0180 (17)	0.0102 (17)	0.0116 (17)
N2	0.0279 (18)	0.031 (2)	0.0265 (18)	0.0080 (16)	0.0069 (15)	0.0093 (15)
C1	0.060 (3)	0.046 (3)	0.039 (3)	0.030 (3)	0.013 (2)	0.018 (2)
C2	0.067 (4)	0.057 (3)	0.053 (3)	0.034 (3)	0.022 (3)	0.036 (3)
C3	0.060 (3)	0.038 (3)	0.069 (4)	0.021 (3)	0.023 (3)	0.027 (3)
C4	0.044 (3)	0.033 (3)	0.049 (3)	0.015 (2)	0.014 (2)	0.014 (2)
C5	0.032 (2)	0.032 (2)	0.032 (2)	0.0137 (19)	0.0112 (19)	0.0095 (19)
C6	0.031 (2)	0.033 (3)	0.030 (2)	0.009 (2)	0.0087 (18)	0.0076 (19)
C7	0.030 (2)	0.032 (2)	0.028 (2)	0.0112 (19)	0.0059 (18)	0.0099 (18)
C8	0.034 (2)	0.034 (2)	0.033 (2)	0.006 (2)	0.0050 (19)	0.011 (2)
C9	0.035 (2)	0.050 (3)	0.030 (2)	0.007 (2)	0.003 (2)	0.014 (2)
C10	0.037 (3)	0.052 (3)	0.040 (3)	0.017 (2)	0.011 (2)	0.018 (2)
C11	0.087 (4)	0.038 (3)	0.044 (3)	0.029 (3)	0.010 (3)	0.015 (2)
C12	0.079 (4)	0.034 (3)	0.029 (2)	0.021 (3)	0.002 (2)	0.004 (2)
C13	0.059 (3)	0.075 (4)	0.065 (4)	0.022 (3)	0.016 (3)	0.047 (3)

### Geometric parameters (Å, º)

**Table:**

Cd1—Cl1	2.6221 (11)	C3—H3	0.9300
Cd1—Cl3	2.6548 (10)	C3—C4	1.375 (7)
Cd1—Cl4	2.6543 (11)	C4—H4	0.9300
Cd1—Cl2	2.6805 (10)	C4—C5	1.385 (6)
Cd1—N1	2.311 (3)	C5—C6	1.463 (6)
Cd1—N2	2.378 (3)	C6—H6	0.9300
Cd2—Cl1	2.5729 (10)	C7—C8	1.388 (6)
Cd2—Cl3^i^	2.7555 (10)	C7—C12	1.375 (6)
Cd2—Cl3^ii^	2.6874 (10)	C8—H8	0.9300
Cd2—Cl4^ii^	2.5165 (11)	C8—C9	1.381 (6)
Cd2—Cl2	2.7074 (11)	C9—H9	0.9300
Cd2—Cl2^i^	2.6404 (10)	C9—C10	1.385 (6)
N1—C1	1.327 (5)	C10—C11	1.369 (6)
N1—C5	1.348 (5)	C10—C13	1.497 (6)
N2—C6	1.281 (5)	C11—H11	0.9300
N2—C7	1.426 (5)	C11—C12	1.376 (6)
C1—H1	0.9300	C12—H12	0.9300
C1—C2	1.377 (6)	C13—H13A	0.9600
C2—H2	0.9300	C13—H13B	0.9600
C2—C3	1.374 (7)	C13—H13C	0.9600
			
Cl1—Cd1—Cl3	93.00 (3)	C7—N2—Cd1	125.1 (2)
Cl1—Cd1—Cl4	176.14 (3)	N1—C1—H1	118.4
Cl1—Cd1—Cl2	86.03 (3)	N1—C1—C2	123.2 (4)
Cl3—Cd1—Cl2	85.58 (3)	C2—C1—H1	118.4
Cl4—Cd1—Cl3	83.17 (3)	C1—C2—H2	120.7
Cl4—Cd1—Cl2	93.13 (3)	C3—C2—C1	118.6 (5)
N1—Cd1—Cl1	100.54 (9)	C3—C2—H2	120.7
N1—Cd1—Cl3	166.29 (9)	C2—C3—H3	120.4
N1—Cd1—Cl4	83.26 (9)	C2—C3—C4	119.3 (5)
N1—Cd1—Cl2	93.16 (9)	C4—C3—H3	120.4
N1—Cd1—N2	72.51 (12)	C3—C4—H4	120.6
N2—Cd1—Cl1	87.37 (8)	C3—C4—C5	118.8 (4)
N2—Cd1—Cl3	110.60 (9)	C5—C4—H4	120.6
N2—Cd1—Cl4	94.46 (8)	N1—C5—C4	122.0 (4)
N2—Cd1—Cl2	162.83 (8)	N1—C5—C6	118.1 (4)
Cl1—Cd2—Cl3^ii^	100.81 (3)	C4—C5—C6	119.9 (4)
Cl1—Cd2—Cl3^i^	176.84 (3)	N2—C6—C5	121.6 (4)
Cl1—Cd2—Cl2^i^	93.06 (3)	N2—C6—H6	119.2
Cl1—Cd2—Cl2	86.45 (3)	C5—C6—H6	119.2
Cl3^ii^—Cd2—Cl3^i^	81.21 (3)	C8—C7—N2	124.5 (4)
Cl3^ii^—Cd2—Cl2	172.42 (3)	C12—C7—N2	117.3 (4)
Cl4^ii^—Cd2—Cl1	94.45 (4)	C12—C7—C8	118.2 (4)
Cl4^ii^—Cd2—Cl3^ii^	85.18 (3)	C7—C8—H8	120.3
Cl4^ii^—Cd2—Cl3^i^	88.13 (3)	C9—C8—C7	119.3 (4)
Cl4^ii^—Cd2—Cl2^i^	172.49 (3)	C9—C8—H8	120.3
Cl4^ii^—Cd2—Cl2	96.53 (3)	C8—C9—H9	118.6
Cl2^i^—Cd2—Cl3^i^	84.37 (3)	C8—C9—C10	122.8 (4)
Cl2^i^—Cd2—Cl3^ii^	93.30 (3)	C10—C9—H9	118.6
Cl2—Cd2—Cl3^i^	91.45 (3)	C9—C10—C13	121.8 (4)
Cl2^i^—Cd2—Cl2	84.04 (3)	C11—C10—C9	116.5 (4)
Cd2—Cl1—Cd1	95.92 (4)	C11—C10—C13	121.7 (5)
Cd1—Cl3—Cd2^iii^	93.50 (3)	C10—C11—H11	119.1
Cd1—Cl3—Cd2^i^	93.98 (3)	C10—C11—C12	121.7 (5)
Cd2^iii^—Cl3—Cd2^i^	98.79 (3)	C12—C11—H11	119.1
Cd2^iii^—Cl4—Cd1	97.59 (4)	C7—C12—C11	121.3 (4)
Cd1—Cl2—Cd2	91.47 (3)	C7—C12—H12	119.3
Cd2^i^—Cl2—Cd1	96.07 (3)	C11—C12—H12	119.3
Cd2^i^—Cl2—Cd2	95.96 (3)	C10—C13—H13A	109.5
C1—N1—Cd1	126.8 (3)	C10—C13—H13B	109.5
C1—N1—C5	118.1 (4)	C10—C13—H13C	109.5
C5—N1—Cd1	114.5 (3)	H13A—C13—H13B	109.5
C6—N2—Cd1	113.0 (3)	H13A—C13—H13C	109.5
C6—N2—C7	121.8 (3)	H13B—C13—H13C	109.5
			
Cd1—N1—C1—C2	−170.1 (4)	C3—C4—C5—C6	177.4 (4)
Cd1—N1—C5—C4	173.0 (3)	C4—C5—C6—N2	−173.4 (4)
Cd1—N1—C5—C6	−6.4 (5)	C5—N1—C1—C2	0.6 (7)
Cd1—N2—C6—C5	−2.2 (5)	C6—N2—C7—C8	−0.1 (6)
Cd1—N2—C7—C8	−177.5 (3)	C6—N2—C7—C12	178.0 (4)
Cd1—N2—C7—C12	0.5 (5)	C7—N2—C6—C5	−179.9 (4)
N1—C1—C2—C3	−1.4 (8)	C7—C8—C9—C10	−0.4 (7)
N1—C5—C6—N2	6.0 (6)	C8—C7—C12—C11	−0.6 (8)
N2—C7—C8—C9	177.5 (4)	C8—C9—C10—C11	2.6 (7)
N2—C7—C12—C11	−178.8 (5)	C8—C9—C10—C13	−176.4 (4)
C1—N1—C5—C4	1.2 (6)	C9—C10—C11—C12	−3.8 (8)
C1—N1—C5—C6	−178.3 (4)	C10—C11—C12—C7	2.9 (9)
C1—C2—C3—C4	0.5 (8)	C12—C7—C8—C9	−0.6 (7)
C2—C3—C4—C5	1.1 (7)	C13—C10—C11—C12	175.2 (5)
C3—C4—C5—N1	−2.0 (7)		

Symmetry codes: (i) −*x*, −*y*, −*z*; (ii) *x*−1, *y*, *z*; (iii) *x*+1, *y*, *z*.
